# Adsorption performance of M-doped (M = Ti and Cr) gallium nitride nanosheets towards SO_2_ and NO_2_: a DFT-D calculation

**DOI:** 10.1039/d0ra03251d

**Published:** 2020-07-24

**Authors:** Hossein Roohi, Nastaran Askari Ardehjani

**Affiliations:** Computational Quantum Chemistry Laboratory, Department of Chemistry, Faculty of Science, University of Guilan Rasht Iran hroohi@guilan.ac.ir +98 131 3233262

## Abstract

The structure, adsorption characteristics, electronic properties, and charge transfer of SO_2_ and NO_2_ molecules on metal-doped gallium nitride nanosheets (M-GaNNSs; M = Ti and Cr) were scrutinized at the Grimme-corrected PBE/double numerical plus polarization (DNP) level of theory. Two types, M_Ga_-GaNNSs and M_N_-GaNNSs, of doped nanostructures were found. The M_Ga_ sites are more stable than the M_N_ sites. The results showed that adsorption of SO_2_ and NO_2_ molecules on Ti_Ga,N_-GaNNSs is energetically more favorable than the corresponding Cr_Ga,N_-GaNNSs. The stability order of complexes is energetically predicted to be as NO_2_–Ti_Ga_-GaNNS > NO_2_–Ti_N_-GaNNS > SO_2_–Ti_Ga_-GaNNS > NO_2_–Cr_N_-GaNNS > SO_2_–Ti_N_-GaNNS > NO_2_–Cr_Ga_-GaNNS > SO_2_–Cr_N_-GaNNS > SO_2_–Cr_Ga_-GaNNS. The electron population analysis shows that charge is transferred from M_Ga,N_-GaNNSs to the adsorbed gases. The Ti_Ga_-GaNNS is more sensitive than the other doped nanostructures to NO_2_ and SO_2_ gases. It is estimated that the sensitivity of Ti_Ga_-GaNNS to NO_2_ gas is more than to SO_2_ gas.

## Abstract 2

The influences of transition metals (Cr and Ti) doping on the adsorption behavior of SO_2_ and NO_2_ gases on the metal doped Gallium Nitride Nanosheet (GaNNS) were explored at Grimme-corrected PBE/double numerical plus polarization (DNP) level of theory.

## Graphical abstract

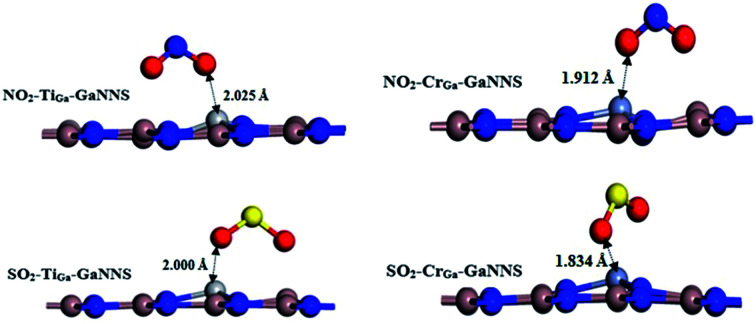

## 1. Introduction

At present, air pollution is a significant factor limiting economic progression.^1^ The emission of toxicant gases into the air is a serious matter due to the dangers of these air pollutants.^2^ The source of air pollutants might be extensive in the Earth’s environment.^3^ Sulfur dioxide (SO_2_) and nitrogen dioxide (NO_2_) are noteworthy gaseous pollutants, discharged from natural and industrial procedures which have major environmental effects.^4–6^ Thus, specific harmful gas detecting will be a major advantage to daily life for all people.^7–9^

For the first time in 2005, boron nitride (BN) nanosheets were forecast.^10^ The honeycomb samples of BN sheets have analogies similar to graphene with equal numbers of alternating boron and nitrogen atoms that exhibit remarkable properties.^11^ The electronic properties of BN sheets can be modified by B or N vacancies, Stone–Wales defects and doping heteroatoms.^12–16^ In recent years, different studies have been done *via* surface quantum engineering of BN nanosheets.^17,18^ For BN modification, the doped BN nanosheets were explored for developing a sensor for detecting harmful gases.^19–21^

Recently, III–V nanostructures have attracted great attention for their potential applications in novel electronic,^22–24^ optical,^25–27^ and electrochemical devices.^28–30^ One of the III–V nanostructures were gallium nitride nanosheets (GaNNSs) which have been theoretically predicted^31–33^ and then experimentally discovered.^34,35^ It was found that the GaNNSs have many remarkable properties such as a high surface area to volume ratio, high thermal stability and a tunable band gap indicating that GaNNSs have advantages in electronic usage such as effective gas sensor applications and so on.^36^ There are some experimental studies focusing on the GaN based NO_2_ and SO_2_ sensors.^37–40^ Bishop *et al.*^41^ suggested a double Schottky junction NO_2_ gas sensor based on BGaN/GaN. Triet *et al.* synthesized Al_0.27_Ga_0.73_N/GaN-based Schottky diode sensors for SO_2_ gas detection.^42^ For example, the adsorption capabilities of gallium nitride nanosheets towards noxious gases (such as HCN, NH_3_, H_2_S, H_2_, CO_2_ and H_2_O) have been described.^43^ Therefore, most of the research studies have focused on nanomaterials for increasing the adsorption of adsorbates on GaNNSs. For this purpose, the electronic properties of GaNNS can be modified by doping which generates more reactive adsorption sites.^44,45^ Transition metals such as Ti, Cr, Fe, Ni and Zn have been theoretically explored as dopants in GaNNSs to increase the adsorption properties towards CO harmful gases.^46^ The adsorption of H_2_S, NH_3_ and SO_2_ molecules on pure and doped GaNNSs has been considered using first-principles calculations. The results show that the metal doped GaNNSs are more suitable for gas molecules detection compared with the pure ones.^47^ The doping effect of metal atoms on the electronic properties of GaNNSs was studied for tuning the optoelectronic properties, gas adsorption, hydrogen storage and catalytic reaction.^48–51^ The electronic and optical properties of GaNNSs as a function of thickness and strain with predictive calculations were scrutinized.^52^ Based on the results reported about the magnetic properties of GaNNSs, the metallic and ferromagnetic properties of GaNNSs can be attained by semi-hydrogenation.^53^ The chemical oxidation of GaNNSs was explored by using first-principles calculations^54^ that show the oxygen adsorption mechanism can be useful for application in novel semiconducting materials. So, it would be attractive to continue investigating the promising applications of GaNNSs in gas sensors.

To the best of our knowledge, this is the first report on the adsorption of SO_2_ and NO_2_ molecules on the surface of Ti and Cr doped GaNNSs. The influence of transition metals doping on the adsorption behavior of SO_2_ and NO_2_ on the metal doped GaNNSs for exploring the possibility of using the doped GaNNSs as candidates for removing and sensing of these molecules was considered herein at the Grimme-corrected PBE/double numerical plus polarization (DNP) level of theory.

## 2. Computational details

In this theoretical research, the double numerical plus polarization (DNP) basis sets were selected implemented in the DMol^3^ package.^55,56^ The periodic spin-unrestricted DFT calculation is employed using generalized-gradient approximation (GGA) with the Perdew–Burke–Ernzerhof (PBE) functional.^57^ The density functional semi-core pseudopotentials (DSPP) were generated by fitting all-electron relativistic DFT results.^58^

To consider the van der Waals (vdW) interactions, an empirical dispersion-corrected density functional theory (DFT-D) was used in the calculations. The Brillouin zone integration was sampled using a 10 × 10 × 1 Monkhorst–Pack grid. A convergence tolerance of energy of 1.0 × 10^−5^ Ha, maximum force of 0.001 Ha per Å and maximum displacement of 0.005 Å were employed in all the geometry optimizations. To get reliable results, the real space global orbital cutoff radius was set as high as 5.2 Å and the smearing of electronic occupations to be 0.005 Ha.

To calculate the adsorption energies (AE) of the SO_2_ and NO_2_ molecules on the pure and metal doped GaNNSs, the following equation is given:

1 AE = *E*_T_ − [*E*_S_ + *E*_m_]

where *E*_T_, *E*_S_ and *E*_m_ are the energies of gas–M-GaNNSs complexes, M-GaNNSs and SO_2_ or NO_2_ molecules, respectively.

## 3. Results and discussion

### 3.1. Adsorption of SO_2_ and NO_2_ gas molecules over pure GaNNSs

**Fig. 1:**
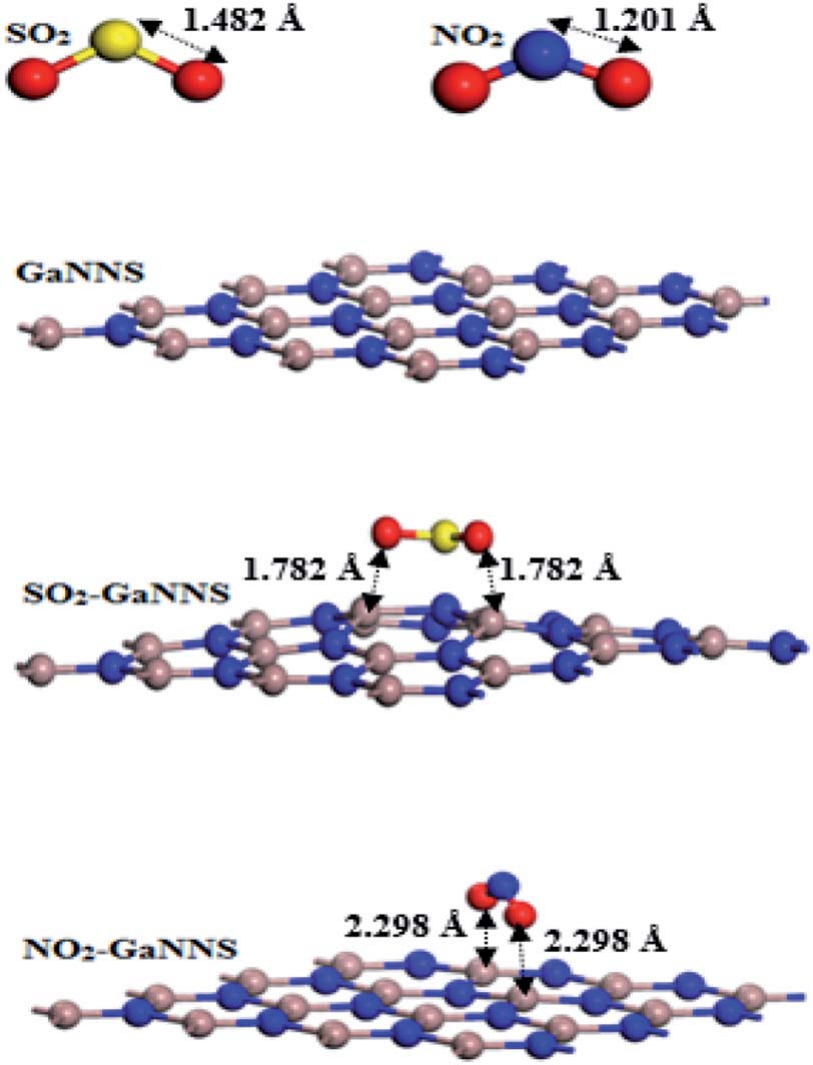
The optimized geometries of SO_2_, NO_2_, pure and most stable adsorption complexes of GaNNSs.

The optimized geometries of adsorbed molecules and gallium nitride nanosheets are displayed in Fig. 1. As demonstrated in Fig. 1, the calculated bond lengths of X=O (X = N and S) in free SO_2_ and NO_2_ molecules are 1.482 Å and 1.201 Å, respectively, and that of the Ga–N bond length in the optimized geometry of GaNNS is 1.861 Å.

To find the most stable complexes obtained from adsorption of SO_2_ and NO_2_ gas molecules on the GaNNS, several configurations of SO_2_ and NO_2_ molecules on top of the GaNNSs are explored. The most stable adsorption complexes are illustrated in Fig. 1. After optimization, the *C*_2_ axis of the SO_2_ molecule is parallel to the GaNNS and that of the NO_2_ molecule is perpendicular to the nanosheet. As presented in Table 1, the nearest distances between the SO_2_ and NO_2_ molecules with SO_2_–GaNNS and NO_2_–GaNNS are 1.782 Å and 2.298 Å, respectively.

**Table 1:** The calculated adsorption energy (AE), equilibrium distance between molecules and nanosheet (*D*), charge of Ga and N atoms (*Q*), charge transfer (CT) and band gaps for the most stable adsorption complexes

Configurations	AE (kcal mol^−1^)	*D* (Å)	*Q* (*e*)	CT (*e*)	Band gap (eV)
Pure GaNNS	—	—	0.39^Ga^ (−0.39)^N^	—	2.52
SO_2_–GaNNS	−27.22	1.782	0.40 (−0.38)	0.17	2.51
NO_2_–GaNNS	−11.86	2.298	0.39 (−0.39)	0.09	0.29

The values of the adsorption energies (AEs) are −27.22 and −11.86 kcal mol^−1^ for SO_2_–GaNNS and NO_2_–GaNNS complexes, in good agreement with the smaller SO_2_–GaNNS distance obtained. Hence, the SO_2_ and NO_2_ molecules are chemically adsorbed on the GaNNSs. The adsorption energy for the SO_2_ molecule on the GaNNS is comparable with those found for graphene (−6.45 kcal mol^−1^)^59^ and boron nitride nanosheet (−7.14 kcal mol^−1^).^21^ For adsorption of NO_2_ on graphene, the calculated adsorption energy is −11.06 kcal mol^−1^.^41^

The Hirshfeld charges on the Ga and N atoms in pristine GaNNS are 0.39*e* and −0.39*e*, respectively, which change to 0.40*e* and −0.38*e* in SO_2_–GaNNS, and 0.39*e* and −0.39*e* in NO_2_–GaNNS. The charges on S and O change from 0.38*e* and −0.19*e* in free SO_2_ to 0.33 *e* and −0.25*e* in SO_2_–GaNNS, respectively. Besides, the charges on N and O are 0.17*e* and −0.09*e* which change to 0.11*e* and −0.10*e* in NO_2_-GaNNS. This reveals that oxygen atoms in SO_2_–GaNNS and NO_2_–GaNNS complexes have the main contribution to charge transfer between the gas and nanosheet. The charge analyses show that the 0.17*e* and 0.09*e* charges are shifted from the GaNNSs to the SO_2_ and NO_2_ molecules, respectively, in good agreement with greater AE found for the SO_2_–GaNNS complex.

The negative AEs demonstrate the orbital interactions between the gases and GaNNS. The Mulliken electron populations of the total and each of the s, p and d orbitals before and after interactions are given in Table 2. Inspection of the s, p and d orbital contributions in the free gases and in the SO_2_–GaNNS and NO_2_–GaNNS complexes indicate that the p orbital of the S atom in SO_2_–GaNNS and O atom in NO_2_–GaNNS have the most contribution in the interaction of molecules with the d orbital of Ga and p orbital of N atoms in the GaNNS. Comparison of the total electron population of orbitals shows that the population increases by 0.352*e* for SO_2_ and 0.169*e* for NO_2_ after interaction of the gas with the surface. This indicates that the adsorbates will get electrons from the GaNNSs. The change in the electronic population of the orbitals in SO_2_–GaNNS is greater than for NO_2_–GaNNS, in good agreement with the greater AE and Hirshfeld charge transfer values found for SO_2_–GaNNS compared with NO_2_–GaNNS.

**Table 2:** Mulliken electron population of the total and each of the s, p and d orbitals before and after interactions

	Total pop.	Orbital				Total pop.	Orbital		
		s	p	d			s	p	d
Free SO_2_					NO_2_–Cr_Ga_-GaNNS				
S	15.558	5.838	8.872	0.848	N	6.72	3.637	2.942	0.14
O	8.22	3.836	4.346	0.04	O	8.346	3.839	4.461	0.047
Free NO_2_					Cr	13.317	2.592	6.454	4.272
N	6.646	3.449	3.028	0.17	NO_2_–Cr_N_-GaNNS				
O	8.176	3.851	4.272	0.055	N	6.759	3.684	2.951	0.124
SO_2_–GaNNS					O	8.343	3.852	4.443	0.05
S	15.514	5.674	8.922	0.918	Cr	13.917	2.71	6.441	4.767
O	8.424	3.846	4.546	0.032	SO_2_–Ti_Ga_-GaNNS				
NO_2_–GaNNS					S	15.613	5.802	9.203	0.608
N	6.669	3.541	2.972	0.156	O	8.364	3.834	4.499	0.031
O	8.25	3.841	4.36	0.049	Ti	11.018	2.433	6.304	2.281
Ti_Ga_-GaNNS					SO_2_–Ti_N_-GaNNS				
Ti	11.244	2.658	6.202	2.384	S	15.651	5.82	9.29	0.542
Ti_N_-GaNNS					O	8.384	3.832	4.522	0.03
Ti	11.465	2.612	6.347	2.505	Ti	11.322	2.51	6.383	2.43
Cr_Ga_-GaNNS					SO_2_–Cr_Ga_-GaNNS				
Cr	13.357	2.655	6.345	4.357	S	15.706	5.81	9.364	0.533
Cr_N_-GaNNS					O	8.399	3.837	4.531	0.03
Cr	14.014	2.888	6.232	4.894	Cr	13.286	2.589	6.463	4.234
NO_2_–Ti_Ga_-GaNNS					SO_2_–Cr_N_-GaNNS				
N	6.72	3.644	2.932	0.142	S	15.6	5.802	9.198	0.6
O	8.4	3.838	4.518	0.042	O	8.423	3.856	4.532	0.034
Ti	11.048	2.45	6.3	2.3	Cr	13.842	2.714	6.462	4.667
NO_2_–Ti_N_-GaNNS									
N	6.734	3.666	2.934	0.134					
O	8.382	3.86	4.478	0.046					
Ti	11.386	2.516	6.44	2.43					

### 3.2. Ti and Cr doped GaNNSs

In order to investigate the effect of metal doping on the geometrical and electronic properties of the GaNNSs, one of the central atoms in the nanosheet was substituted by Ti and Cr metal atoms. Hereafter, M_Ga_-GaNNS and M_N_-GaNNS denote that Ga and N atoms in GaNNS have been substituted by M metal atoms, respectively.

**Fig. 2:**
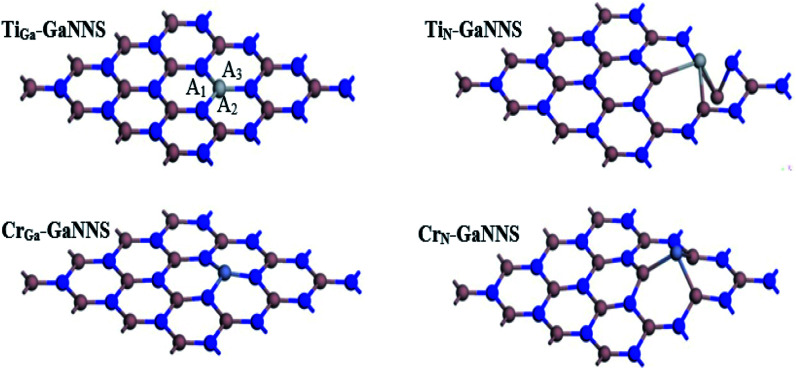
Optimized geometries of Ti and Cr doped GaNNSs.

The optimized structures of M_N(Ga)_-GaNNSs are illustrated in Fig. 2. The average bond distances between the metal atoms and the neighboring atoms are given in Table 3. The results show that the M–Ga bonds in M_N_-GaNNS nanostructures are longer than the M–N bonds in M_Ga_-GaNNSs. For example, the Ti–Ga and Cr–Ga bonds are longer than the Ti–N and Cr–N bonds by about 0.92 and 0.62 Å, respectively. Accordingly, it is predicted that binding of the metal to the nanosheet is stronger for M_Ga_-GaNNS than M_N_-GaNNS. Fig. 2 presents the three bond angles *A*_1_, *A*_2_ and *A*_3_ around the M atoms of the NS and their average values are listed in Table 3. The averages of the three bond angles are 120.0°, 119.9°, 83.1° and 87.1° in Ti_Ga_-GaNNS, Cr_Ga_-GaNNS, Ti_N_-GaNNS and Cr_N_-GaNNS, respectively.

**Table 3:** The calculated average of M–N and M–Ga bond distances, average of bond angles (*A*_1_, *A*_2_ and *A*_3_), binding energies of pristine and M (M = Ti and Cr) doped GaNNSs and Hirshfeld charge values of M atoms

Configurations	Bond distances (Å)	Bond angle (°)	Binding energies (kcal mol^−1^)	*Q* (*e*)
GaNNS	1.862	118.2	—	—
Ti_Ga_-GaNNS	1.913	120.0	−330.5	0.30
Ti_N_-GaNNS	2.838	83.1	−236.1	0.26
Cr_Ga_-GaNNS	1.883	119.9	−246.9	0.43
Cr_N_-GaNNS	2.505	87.1	−61.6	0.28

The binding energies (BEs) of Ti_Ga_-GaNNS, Ti_N_-GaNNS, Cr_Ga_-GaNNS and Cr_N_-GaNNS are −330.5, −236.1, −246.9 and −61.6 kcal mol^−1^, respectively (Table 3). It is found that the BEs increase in the order M_Ga_-GaNNSs > M_N_-GaNNSs so that the value for the Ti_Ga_-GaNNS structure is greater than other ones. Therefore, the Ga sites for M doping are energetically more appropriate than N sites. The sequence of BEs for the M_Ga_-GaNNS series is Ti_Ga_ > Cr_Ga_ and that of the M_N_-GaNNS series is Ti_N_ > Cr_N_. The more-negative BE indicates that the adatom is easier to be incorporated into the GaNNS and M doped GaNNSs are stable.

The Hirshfeld^44^ charge values of M atoms for M-GaNNSs are shown in Table 3. The results show that the charge of the M atom in all M-doped GaNNSs is positive, indicating that the GaNNS in many cases acts as an electron-withdrawing support. The considerable electron transfer from the metal atom to the GaNNS leads to the strong bonding between the M atom and its neighbor atoms and stabilization of single-metal doped GaNNS. Besides, the positive charge of the M atom in M_Ga_-GaNNSs is more than M_N_-GaNNSs, in good agreement with their binding energy (BE) and the average value of the M–NS bond length. Thus, because of greater transfer of charge between the nanosheet and M atoms in M_Ga_-GaNNSs with respect to the corresponding M_N_-GaNNSs, the M_Ga_-GaNNSs are predicted to be more stable than M_N_-GaNNSs.

### 3.3. Adsorption of SO_2_ and NO_2_ gas molecules over Ti-doped GaNNSs

**Fig. 3:**
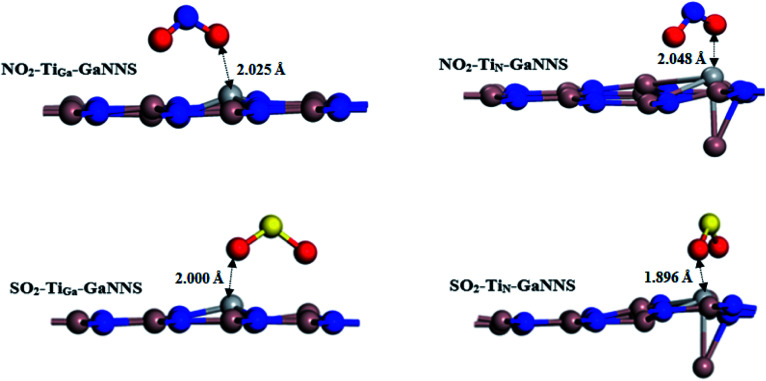
The most stable adsorption configurations of SO_2_ or NO_2_ on Ti-doped GaNNSs.

Now, we investigate the adsorption of SO_2_ and NO_2_ gas molecules on the Ti-doped GaNNSs as displayed in Fig. 3. Our results show that for the SO_2_–Ti_Ga_-GaNNS and NO_2_–Ti_Ga_-GaNNS complexes, the averages of the three binding distances (Ti–N) are 1.901 Å and 1.899 Å, respectively. For the SO_2_–Ti_N_-GaNNS and NO_2_–Ti_N_-GaNNS complexes, the averages of the three binding distances (Ti–Ga) are 2.983 Å and 2.949 Å, respectively. The calculated averages of the bond lengths of the S–O and N–O bonds in SO_2_–Ti_Ga_-GaNNS, SO_2_–Ti_N_-GaNNS, NO_2_–Ti_Ga_-GaNNS and NO_2_–Ti_N_-GaNNS have increased from 1.482 and 1.201 Å to 1.576, 1.606, 1.274 and 1.288 Å, respectively. These results show that the change in the SO_2_ and NO_2_ bond lengths upon adsorption on the Ti_N_-GaNNS is greater than those of Ti_Ga_-GaNNS.

**Table 4:** Adsorption energy (AE), the shortest equilibrium distance between molecules and nanosheet (*D*) and Hirshfeld charge transfer (CT) for the most stable configurations of SO_2_ and NO_2_ on metal doped GaNNSs

Configurations	AE (kcal mol^−1^)	*D* (Å)	CT^a^ (*e*)
**NO_2_**			0.00
NO_2_–Ti_Ga_-GaNNS	−76.83	2.025	0.21
NO_2_–Ti_N_-GaNNS	−70.68	2.048	0.25
NO_2_–Cr_Ga_-GaNNS	−54.79	1.912	0.22
NO_2_–Cr_N_-GaNNS	−60.81	1.952	0.31
**SO_2_**			
SO_2_–Ti_Ga_-GaNNS	−61.06	2.000	0.16
SO_2_–Ti_N_-GaNNS	−58.36	1.896	0.21
SO_2_–Cr_Ga_-GaNNS	−40.46	1.834	0.27
SO_2_–Cr_N_-GaNNS	−53.28	2.124	0.26

a Absolute value of the sum of atomic charges in complexed gases.

The modified surface of Ti-doped GaNNSs facilitates the doped region to interact with approaching SO_2_ and NO_2_ molecules because of the higher chemical reactivity of the doped M atom. The results show that the SO_2_⋯Ti distance of SO_2_–Ti_Ga_-GaNNS complex is larger than SO_2_–Ti_N_-GaNNS ones. Also, the NO_2_⋯Ti distance for the NO_2_–Ti_N_-GaNNS complex is larger than for NO_2_–Ti_Ga_-GaNNS, indicating that the interaction in these complexes is stronger than in other ones.

The range of adsorption energies for SO_2_ and NO_2_ adsorbed on Ti-doped GaNNSs was between −58.36 to −61.06 and −70.68 to −76.83 kcal mol^−1^, respectively. The negative value of the AE indicates that adsorption of SO_2_ and NO_2_ on Ti doped GaNNSs is an exothermic process. It is found that SO_2_ and NO_2_ molecules are adsorbed on Ti_N_-GaNNSs in the sequence NO_2_–Ti_N_-GaNNS > SO_2_–Ti_N_-GaNNS and on the Ti_Ga_-GaNNSs in the order NO_2_–Ti_Ga_-GaNNS > SO_2_–Ti_Ga_-GaNNS. Besides, it is found that the SO_2_ and NO_2_ adsorption energy values on Ti_Ga_-GaNNSs are greater than on Ti_N_-GaNNSs. The obtained results indicate that the adsorption capability of Ti_Ga_-GaNNSs is greater than that of Ti_N_-GaNNSs. Our results show that SO_2_ and NO_2_ molecules are chemically adsorbed on all Ti_Ga,N_-GaNNSs.

The Hirshfeld population analysis presents that the charges are transferred from the Ti_Ga,N_-GaNNSs complexes to the SO_2_ and NO_2_ molecules. In other words, SO_2_ and NO_2_ act as electron acceptors. There is a correlation between charge transfer values and adsorption energy in the process of adsorption of SO_2_ and NO_2_ on the GaNNSs. Comparison of charge transfer values between M-doped GaNNSs and SO_2_ and NO_2_ molecules demonstrate that the value for adsorption of NO_2_ is greater than that of the corresponding SO_2_ one, with the exception of that obtained for Cr_Ga_-GaNNS. This indicates that other parameters than charge transfer are responsible for the stability of the complexes.

The electron populations of orbitals given in Table 2 show that population of d-orbitals as well as total population of the M metals decrease upon the interaction of gases with Ti_Ga,N_-GaNNSs. Besides, total electron population of orbitals in gases increases after adsorption of gases on the surface. This finding reveals that gases will take the electrons from Ti_Ga,N_-GaNNSs. After adsorption of gases, total electron populations of orbitals of NO_2_ (0.522*e* for NO_2_–Ti_Ga_-GaNNS and 0.500*e* for NO_2_–Ti_N_-GaNNS) are greater than those of SO_2_ (0.343*e* for SO_2_–Ti_Ga_-GaNNS and 0.421*e* for SO_2_–Ti_N_-GaNNS), in good agreement with the greater AEs found for NO_2_–Ti_Ga,N_-GaNNS complexes. The decrease in total electron population of Ti before and after interaction is −0.196*e* and −0.079*e* in NO_2_–Ti_Ga_-GaNNS and NO_2_–Ti_N_-GaNNS, respectively, in good agreement with the greater AE found for NO_2_–Ti_Ga_-GaNNS. Besides, the total electron population of Ti after interaction with SO_2_ decreases by −0.226*e* and −0.143*e* in SO_2_–Ti_Ga_-GaNNS and SO_2_–Ti_N_-GaNNS, respectively, in good agreement with the greater AE found for SO_2_–Ti_Ga_-GaNNS.

### 3.4. Adsorption of SO_2_ and NO_2_ gas molecules over Cr-doped GaNNSs

**Fig. 4:**
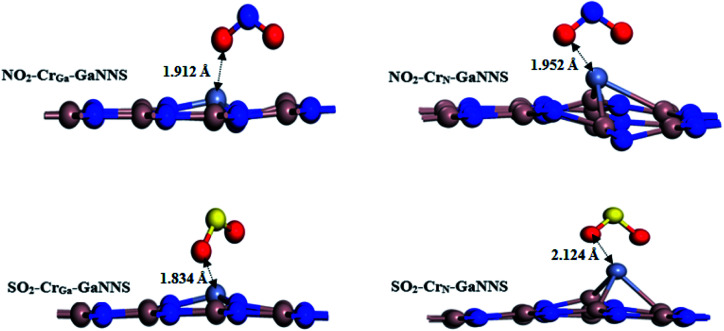
The most stable adsorption configurations of SO_2_ or NO_2_ on Cr-doped GaNNSs.

For the SO_2_–Cr_Ga_-GaNNS and NO_2_–Cr_Ga_-GaNNS complexes, the averages of three binding distances (Ti–N) are 1.834 Å and 1.912 Å, respectively. The calculated averages of bond lengths of S–O and N–O bonds in SO_2_–Cr_Ga_-GaNNS, SO_2_–Cr_N_-GaNNS, NO_2_–Cr_Ga_-GaNNS and NO_2_–Cr_N_-GaNNS have increased from 1.482 Å to 1.527, 1.574, 1.277 and 1.307 Å, respectively. For the SO_2_–Cr_N_-GaNNS and NO_2_–Cr_N_-GaNNS complexes, the averages of the three binding distances (Ti–Ga) are 2.124 Å and 1.952 Å, respectively. These results show that the change in the SO_2_ and NO_2_ bond lengths upon adsorption on the Cr_N_-GaNNS is greater than that for Cr_Ga_-GaNNS. The optimized structures of the NO_2_ and SO_2_ adsorbed on Cr-doped GaNNSs are illustrated in Fig. 4. The modified surface of the Cr-doped GaNNSs facilitates the doped region to interact with approaching SO_2_ and NO_2_ molecules because of the higher chemical reactivity of the doped M atom. The results show that the SO_2_⋯Cr distance for the SO_2_–Cr_N_-GaNNS complex is larger than that for the SO_2_–Cr_Ga_-GaNNS one. Also, the NO_2_⋯Cr distance for the NO_2_–Cr_N_-GaNNS complex is larger than that for NO_2_–Cr_Ga_-GaNNS, indicating that interaction in these complexes is stronger than for other ones.

The adsorption energies for SO_2_ adsorbed on Cr_Ga_-GaNNS and Cr_N_-GaNNS are −40.46 and −53.28 kcal mol^−1^ and those for NO_2_ are −54.79 and −60.81 kcal mol^−1^, respectively. The negative value of the AE indicates that adsorption of SO_2_ and NO_2_ on Cr doped GaNNSs is an exothermic process. It is found that the ability of Cr_N_-GaNNS towards adsorption of SO_2_ and NO_2_ molecules is in the sequence NO_2_–Cr_N_-GaNNS > SO_2_–Cr_N_-GaNNS and that of Cr_Ga_-GaNNS is in the order NO_2_–Cr_Ga_-GaNNS > SO_2_–Cr_Ga_-GaNNS. In addition, it is found that SO_2_ and NO_2_ adsorption energies on Cr_N_-GaNNS are greater than on Cr_Ga_-GaNNS. The obtained results indicate that the adsorption capability of Cr_N_-GaNNS is greater than Cr_Ga_-GaNNS. Our results show that SO_2_ and NO_2_ molecules are chemically adsorbed on all M-doped GaNNSs.

The Hirshfeld population analysis shows that the charges are transferred from the Cr_Ga,N_-GaNNS complexes to the SO_2_ and NO_2_ molecules. As can be seen in Table 4, the CT values are 0.22*e*, 0.31*e*, 0.27*e* and 0.26*e* in NO_2_–Cr_Ga_-GaNNS, NO_2_–Cr_N_-GaNNS, SO_2_–Cr_Ga_-GaNNS and SO_2_–Cr_N_-GaNNS, respectively. This finding reveals that the charge transferred from the nanosheet to the gas in NO_2_–Cr_N_-GaNNS is greater than in other complexes, in good agreement with the greater AE obtained for these complexes. It should be noted that other parameters than charge transfer can affect the adsorption of gases.

Analysis of the electron population of orbitals involved in the interaction between gases and nanosheets given in Table 2 reveals that the total electron population of Cr decreases by −0.040*e*, −0.097*e*, −0.071*e* and −0.172*e* in NO_2_–Cr_Ga_-GaNNS, NO_2_–Cr_N_-GaNNS, SO_2_–Cr_Ga_-GaNNS, SO_2_–Cr_N_-GaNNS, respectively, in good agreement with the greater AE found for NO_2_(SO_2_)–Cr_N_-GaNNSs compared with NO_2_(SO_2_)–Cr_Ga_-GaNNSs. In addition, the results show that the population of d-orbitals of Cr decreases and those of the NO_2_ and SO_2_ gases increase upon interaction with Cr_Ga,N_-GaNNSs. This finding demonstrates that gases will get electrons from Cr_Ga,N_-GaNNSs, in good agreement with the computed Hirshfeld charge transfer from the surface to adsorbed gases.

### 3.5. HOMO and LUMO based electronic properties

There is an obvious difference between the electronic properties of doped and un-doped GaNNSs. As can be seen in Table 5, compared to pristine GaNNS, the energy of the highest occupied molecule orbital (HOMO) increases and that of the lowest un-occupied molecular orbital (LUMO) decreases in doped GaNNS so that the amount of increase in HOMO is greater than decrease in LUMO. After adsorption of NO_2_, the energies of the HOMO and LUMO decrease, but the LUMO energy shows a further decrease. In the case of SO_2_, adsorption of gas on the Ti-doped GaNNS decreases both the HOMO and LUMO, but its adsorption on the Cr-doped GaNNS increases them. These changes in HOMO and LUMO energy levels lead to a change in the HOMO–LUMO gap and, in turn, in the electronic properties of GaNNS.

**Table 5:** The *E*_HOMO_, *E*_LUMO_, the HOMO–LUMO gap and gap change of pure and NO_2_ or SO_2_ on M doped GaNNSs

Configurations	*E*_HOMO_ (eV)	*E*_LUMO_ (eV)	*E*_g_ (eV)	Δ*E*_g_ (eV)
GaNNS	−5.75	−3.23	2.52	—
Ti_Ga_-GaNNS	−3.76	−3.28	0.47	2.05
Ti_N_-GaNNS	−4.50	−3.60	0.90	1.62
Cr_Ga_-GaNNS	−4.28	−3.64	0.64	1.88
Cr_N_-GaNNS	−4.26	−3.78	0.47	2.05
NO_2_–GaNNS	−5.83	−5.53	0.29	2.23
NO_2_–Ti_Ga_-GaNNS	−5.90	−3.62	2.28	1.81
NO_2_–Ti_N_-GaNNS	−4.96	−4.01	0.95	0.05
NO_2_–Cr_Ga_-GaNNS	−4.52	−3.87	0.65	0.01
NO_2_–Cr_N_-GaNNS	−5.08	−4.18	0.91	0.44
SO_2_–GaNNS	−5.90	−3.37	2.11	0.41
SO_2_–Ti_Ga_-GaNNS	−4.69	−3.29	1.39	0.92
SO_2_–Ti_N_-GaNNS	−4.59	−3.47	1.11	0.21
SO_2_–Cr_Ga_-GaNNS	−3.45	−2.91	0.53	0.11
SO_2_–Cr_N_-GaNNS	−3.82	−3.53	0.28	0.19

The results indicate that the band gap energies in both M_Ga,N_ doped GaNNSs are smaller than the pure GaNNS, making it more conductive. The results given in Table 5 demonstrate that the band gap value of pristine GaNNS is 2.52 eV that changes to 2.11 eV in SO_2_–GaNNS and 0.29 eV in NO_2_–GaNNS. Because of the greater decrease in the LUMO level in NO_2_–GaNNS with respect to that of SO_2_–GaNNS, the change in the band gap for SO_2_–GaNNS is lesser than for NO_2_–GaNNS. Therefore, it is predicted that GaNNS is more sensitive to NO_2_ gas than SO_2_ gas. So, the large changes in band gap value for GaNNS (2.52 to 0.29 eV) with NO_2_ gas molecule adsorption can lead to a significant change in electrical conductivity.

The electronic properties of doped GaNNSs are affected by the adsorption of SO_2_ and NO_2_ molecules. Upon adsorption of SO_2_ and NO_2_ molecules on the Ti_Ga,N_-GaNNS complexes the energy gap decreases in comparison with the pristine GaNNS. The energy gap values are in the sequences SO_2_–Ti_N_-GaNNS (1.11 eV) > NO_2_–Ti_N_-GaNNS (0.95 eV) and NO_2_–Ti_Ga_-GaNNS (2.28 eV) > SO_2_–Ti_Ga_-GaNNS (1.39 eV). Reduction of the band gap for SO_2_–Ti_N_-GaNNS is more than for NO_2_–Ti_N_-GaNNS and that for NO_2_–Ti_Ga_-GaNNS is more than for SO_2_–Ti_Ga_-GaNNS. Therefore, it can be concluded that the sensitivity of Ti_N_-GaNNS to SO_2_ gas is greater than to NO_2_ gas. Besides, for Ti_Ga_-GaNNS, it is predicted that the sensitivity to NO_2_ gas is more than to SO_2_ gas. It is a well-known issue that reduction of the energy gap enhances the electrical conductivity. Thus, the electrical conductivities are predicted to be in the order SO_2_–Ti_N_-GaNNS > NO_2_–Ti_N_-GaNNS and NO_2_–Ti_Ga_-GaNNS > SO_2_–Ti_Ga_-GaNNS.

Also, after adsorption of SO_2_ and NO_2_ molecules on the Cr_Ga,N_-GaNNS complexes the band gap decreases in comparison with the pristine GaNNS. The band gap values are in sequence NO_2_–Cr_N_-GaNNS (0.91 eV) > SO_2_–Cr_N_-GaNNS (0.28 eV) and NO_2_–Cr_Ga_-GaNNS (0.65 eV) > SO_2_–Cr_Ga_-GaNNS (0.53 eV). The reduction in band gap for SO_2_–Cr_Ga_-GaNNS is more than for NO_2_–Cr_Ga_-GaNNS and that for NO_2_–Cr_N_-GaNNS is more than for SO_2_–Cr_N_-GaNNS. In consequence, the conductivities of SO_2_–Cr_Ga_-GaNNS and NO_2_–Cr_N_-GaNNS are greater than NO_2_–Cr_Ga_-GaNNS and SO_2_–Cr_N_-GaNNS, respectively. Also, from the changes in band gap energy values, it is forecast that the sensitivity of Cr_N_-GaNNS to NO_2_ gas is more than to SO_2_ gas.

**Fig. 5:**
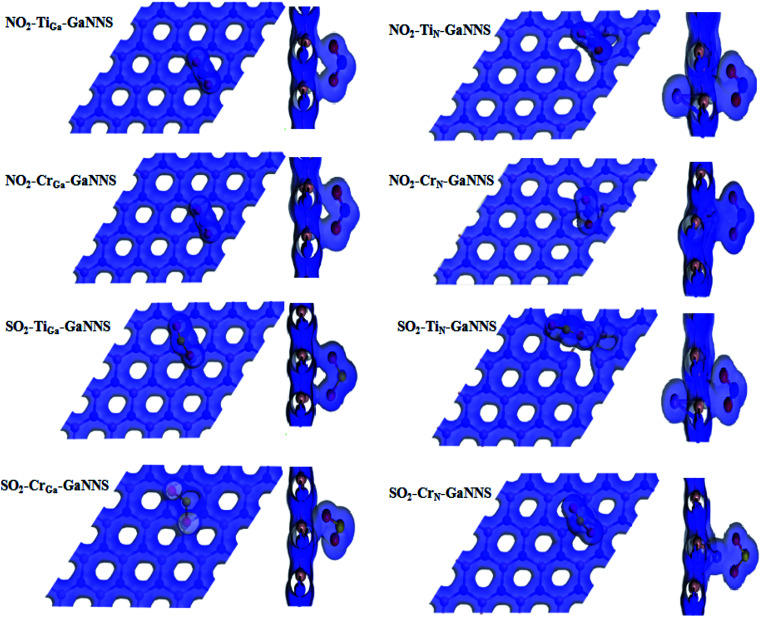
The electron density schema (top and side views) of SO_2_ or NO_2_ adsorbed on doped GaNNSs with isovalue = 0.2*e* Å^−3^.

The top and side view plots of the electron density are illustrated in Fig. 5. An orbital overlap can be observed between the NO_2_ (SO_2_) gas molecules and the GaN sheets, revealing the occurrence of a strong chemisorption. It is clearly displayed that the electrons dominantly accumulate in the region between gases and M-GaNNSs. These results are in accordance with the obtained adsorption energies and binding distances. The orbital mixing and the charge transfer are expected to bring significant changes to the electronic structure of the GaN nanosheets which is beneficial for sensing applications. The isovalue for adsorption of gas molecules on GaNNS is 0.2*e* Å^−3^.

**Fig. 6:**
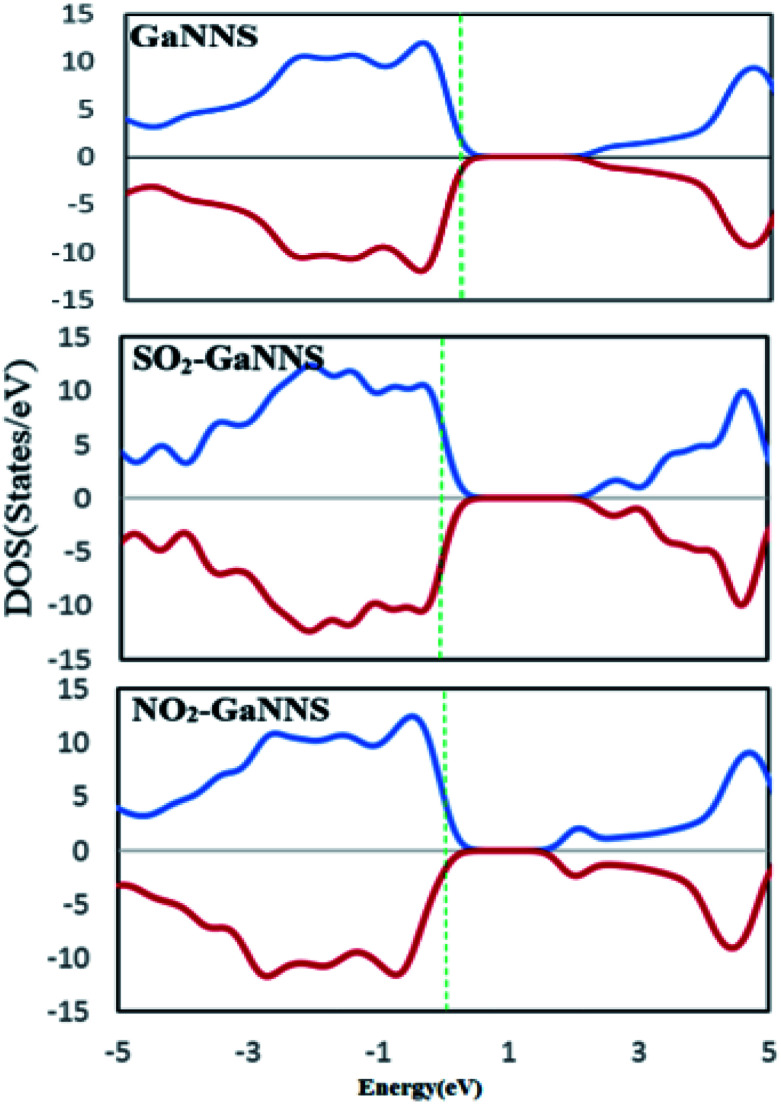
The densities of states (DOSs) of GaNNS and SO_2_ (NO_2_) adsorbed on pristine GaNNSs.

**Fig. 7:**
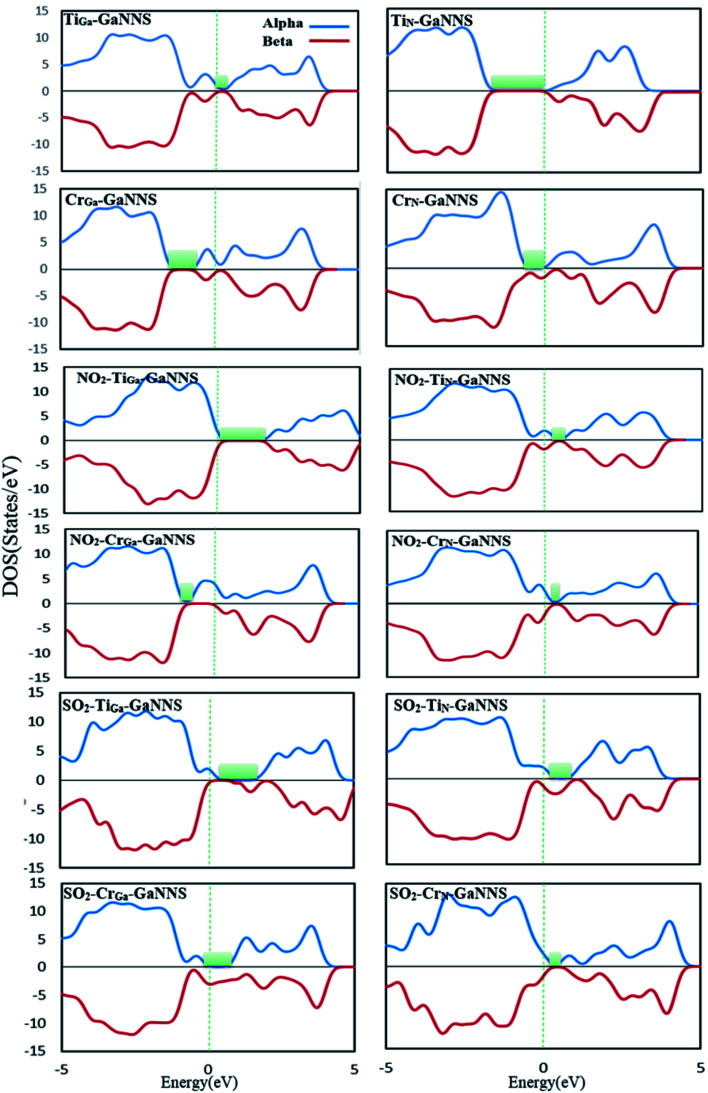
The densities of states (DOSs) of SO_2_ or NO_2_ adsorbed on doped GaNNSs.

To obtain a better understanding about the electronic properties of the complexes, densities of states (DOSs) of the nanostructures are calculated and visualized in Fig. 6 and 7. As can be observed, the GaNNS, NO_2_–GaNNS, SO_2_–GaNNS, NO_2_–Ti_Ga_-GaNNS, NO_2_–Ti_N_-GaNNS and NO_2_–Cr_N_-GaNNS nanostructures are nonmagnetic systems because the spin-up and spin-down in the DOS plots are the same as each other. From this figure, it can be found that new states appear in the band gap regions of NO_2_–Cr_Ga_-GaNNS, SO_2_–Ti_Ga_-GaNNS, SO_2_–Ti_N_-GaNNS, SO_2_–Cr_Ga_-GaNNS and SO_2_–CrN-GaNNS. Since the spin-up and spin-down DOSs are different, NO_2_–Cr_Ga_-GaNNS, SO_2_–Ti_Ga_-GaNNS, SO_2_–Ti_N_-GaNNS, SO_2_–Cr_Ga_-GaNNS and SO_2_–Cr_N_-GaNNS have magnetic properties.

## 4. Conclusions

DFT calculations are performed to consider the adsorption of sulfur dioxide and nitrogen dioxide molecules on metal-doped gallium nitride nanosheets. The results present that adsorption of NO_2_ on M_N_-GaNNS and M_Ga_-GaNNS is energetically more favorable than that of SO_2_ on corresponding NSs. A brief comparison of AEs of SO_2_ and NO_2_ molecules on Ti and Cr doped GaNNSs indicates that the AEs of NO_2_ and SO_2_ on Ti_Ga,N_-GaNNSs are greater than on Cr_Ga,N_-GaNNSs. The electron populations of orbitals were calculated before and after interaction. There is a correlation between the change in electron population of orbitals of adsorbate and adsorbent and the AE obtained for complexes. The electron population analysis shows that charge is transferred from M_Ga,N_-GaNNSs to the adsorbed gases. After the adsorption of SO_2_ and NO_2_ molecules, the electronic properties of the pure and doped GaNNSs indicate the considerable changes in the conductivity of the nanosheets. Furthermore, the sensitivity of Ti_Ga_-GaNNS is predicted to be more than for other NSs toward SO_2_ gas. It is estimated that the sensitivity of Ti_Ga_-GaNNS to NO_2_ gas is more than to SO_2_ gas. Therefore, these results show that GaNNS-based materials can be used as noxious gas sensors.

## Conflicts of interest

The all authors declare that they have no conflict of interest.

## Supplementary Material
